# Medical Epitome Series

**Published:** 1907-04

**Authors:** 


**Medical Epitome Series.** Edited by **Victor C. Pederson,** M.D., Lecturer in surgery in the New York Polyclinic Medical School and Hospital. Pathology, by **John Stenhouse,** M.D., and **John Ferguson,** M.D. 12 mo. pp. 285. Illustrated. Philadelphia and New York: Lea Bros. & Co. (Price, $1.00).

This is the twentieth volume of the Medical Epitome Series published by Lea Brothers and Company, and it marks the near completion of the series, which will number 23 books. The remaining three, covering gynecology, hygiene, and nose and thoat, will be issued shortly. In this volume the authors have devoted the first half of the work to general pathology; the balance is given to the special pathology of the various organs and systems. The arrangement has been made to conform to the latest methods and the work is essentially what it is intended to be,—a masterpiece of epitomisation of a most important subject; so well, so carefully has the work been done that any student is otherwise deficient who cannot lay a firm foundation for further and advanced study by its conscientious use. The book is compact and complete and being intelligent needs no other comment or commendation.

